# Role of thymosin α1 in restoring immune response in immunological nonresponders living with HIV

**DOI:** 10.1186/s12879-024-08985-y

**Published:** 2024-01-17

**Authors:** Chaoyu Chen, Jiangrong Wang, Jingna Xun, Xinyu Zhang, Li Liu, Zichen Song, Renfang Zhang, Jun Chen, Hongzhou Lu

**Affiliations:** 1 Shanghai Public Health Clinical Center, Fudan University, Caolang Road 2901, Jinshan, Shanghai, 201508 China; 2 National Clinical Research Centre for Infectious Diseases, The Third People’ s Hospital of Shenzhen, The Second Affiliated Hospital of Southern University of Science and Technology, Shenzhen, 518112 China

**Keywords:** HIV, Immunological nonresponders, Thymosin α1, Immune response, Thymic output

## Abstract

**Background:**

Immunological nonresponders (INRs) living with HIV are at increased risk of co-infection and multiple tumors, with no effective strategy currently available to restore their T-cell immune response. This study aimed to explore the safety and efficacy of thymosin α1 in reconstituting the immune response in INRs.

**Methods:**

INRs with CD4 + T cell counts between 100 and 350 cells/μL were enrolled and received two-staged 1.6 mg thymosin α1 subcutaneous injections for 24 weeks (daily in the first 2 weeks and biweekly in the subsequent 22 weeks) while continuing antiretroviral therapy. T cell counts and subsets, the expression of PD-1 and TIM-3 on T cells, and signal joint T cell receptor excision circles (sjTREC) at week 24 were evaluated as endpoints.

**Results:**

Twenty three INRs were screened for eligibility, and 20 received treatment. The majority were male (19/20), with a median age of 48.1 years (interquartile range: 40.5–57.0) and had received antiretroviral therapy for 5.0 (3.0, 7.3) years. Multiple comparisons indicated that CD4 + T cell count and sjTREC increased after initiation of treatment, although no significant differences were observed at week 24 compared to baseline. Greatly, levels of CD4 + T cell proportion (17.2% vs. 29.1%, *P* < 0.001), naïve CD4 + and CD8 + T cell proportion (17.2% vs. 41.1%, *P* < 0.001; 13.8% vs. 26.6%, *P* = 0.008) significantly increased. Meanwhile, the proportion of CD4 + central memory T cells of HIV latent hosts (42.7% vs. 10.3%, *P* < 0.001) significantly decreased. Moreover, the expression of PD-1 on CD4 + T cells (14.1% vs. 6.5%, *P* < 0.001) and CD8 + T cells (8.5% vs. 4.1%, *P* < 0.001) decreased, but the expression of TIM-3 on T cellsremained unaltered at week 24. No severe adverse events were reported and HIV viral loads kept stable throughout the study.

**Conclusions:**

Thymosin α1 enhance CD4 + T cell count and thymic output albeit as a trend rather than an endpoint. Importantly, it improves immunosenescence and decreases immune exhaustion, warranting further investigation.

**Trial registration:**

This single-arm prospective study was registered with ClinicalTrials.gov (NCT04963712) on July 15, 2021.

**Supplementary Information:**

The online version contains supplementary material available at 10.1186/s12879-024-08985-y.

## Background

Human immunodeficiency virus type 1 (HIV-1) infection leads to progressive depletion of CD4 + T cells and functional impairment in people living with HIV (PLWH). The introduction of antiretroviral therapy (ART) has reduced morbidity and mortality in PLWH by suppressing viral replication and promoting CD4 + T cell recovery [1, 2]. However, approximately 20% of PLWH fail to restore CD4 + T cell counts despite persistent viral suppression with ART, leading to a heightened susceptibility to opportunistic infections, non-AIDS-related diseases, and deteriorating quality of life as compared to immunological responders (IRs) who achieve substantial CD4 + T cell count recovery [3–5]. Currently, no definitive pharmaceutical intervention exists to restore CD4 + T cell count in INRs. The failure to restore CD4 + T cell count following ART has been attributed primarily to a defect in recent thymic immigrants [6]. A cross-sectional study found INRs maintain a lower proportion of thymic immigrant CD4 + T cells and naïve CD4 + T cells, but a higher proportion of effector memory CD4 + T cells and elevated levels of thymic immigrant CD4 + T cell pyroptosis compared with IRs [7]. Therefore, immunomodulatory agents may hold promise for enhancing thymic recovery and immune reconstitution in INRs.

Thymosin α1 (Tα1), a synthetic thymic polypeptide homologous to a natural product isolated from thymosin fraction 5 of calf thymuses, has emerged as a potential candidate [8]. As an agonist of Toll-like receptors 2 and 9, Tα1 can boost thymic output, stimulate various immune cells populations including dendritic cells, natural killer cells, B cells, and T cells, and promote their proliferation and adaptive response crucial for combatin viral, bacterial, and fungal infections, and cancers [9]. After the advent of the first antiretroviral drug, Tα1 was found to elevate CD4 + T cell levels only in combination with IFN-α and zidovudine [10]. However, a contemporaneous study reporte that IL-2 led to significant increases in CD4 + T cells in the presence of zidovudine but does not when combined with Tα1 [11]. With the popularization of ART, a phase II randomized, controlled open-label trial noted changes primarily in signal joint T cell receptor excision circles (sjTREC) levels in peripheral blood mononuclear cells (PBMCs) but not in CD4 + , CD8 + , and CD45RA + T lymphocyte subsets after week 12 of Tα1 treatment in PLWH [12]. Previous investigations have predominantly focused on antiretroviral-naïve PLWH and considered Tα1 as an adjunct to ART. Here we would like to expand the applicability of Tα1 to explore its potential in immune reconstitution in INRs after long-term ART.

The present prospective pilot trial seeks to assess the safety and efficacy of Tα1 in restoring immune response in INRs. After Tα1 treatment, within 24 weeks, parameters related to thymic output, T cell subsets, immune exhaustion, and immunosenescence were evaluated to characterize the immunological profiles of these participants.

## Methods

### Participants

From September 2021 to October 2021, INRs meeting the inclusion criteria were enrolled at Shanghai Public Health Clinical Center, China. Specifically, all volunteers were between 18 to 65 years old, with HIV infection confirmed by positive ELISA, and achieved viral suppression (HIV RNA < 50 copies/mL) for at least two years following ART. Participants with CD4 + T cell counts between 100 to 350 cells/µL and without active opportunistic infection were eligible for the study. Any cases of allergy to Tα1, pregnancy, drug abuse, non-AIDS related tumors, severe cardiac or central nervous diseases, organ transplantation, and immunotherapies were excluded. Written informed consent was obtained from all participants.

### Design and procedures

The study was a single-arm trial with a longitudinal design. In the first two weeks, each participant got a daily subcutaneous injection of 1.6 mg Tα1, followed by a transition to twice-weekly injections for the subsequent 22 weeks. Following the guidance of the attending physician, participants continued original ART regimens throughout the study period. Zadaxin® (Sci Clone Pharmaceuticals Inc., Shanghai, China) was supplied as a lyophilized powder in vials with 1.6 mg of Tα1, 50 mg mannitol, and sodium phosphate buffer to adjust the pH to 6.8. The powder was reconstituted with sterile water (1 mL) for injection. At week 0 (baseline), 4, 8, 12, and 24, peripheral venous blood (PVB) samples were obtained from each participant for routine laboratory tests. An additional 10 mL PVB was collected for extraction of PBMCs and successive immunological assays. In this study, the primary endpoint was the change in CD4 + T cell count and CD4/CD8 T-cell ratio from baseline to week 24. The secondary endpoints were changes in T cell counts and subsets, proportions of immune exhausted T cells expressed PD-1 and TIM-3, and PBMC sjTREC at each visit.

### Flow cytometry

Flow cytometry (FACS Calibur, BD, USA) was used to measure CD4 + and CD8 + T cell counts immediately after PVB isolation. PBMCs were isolated from PVB using Ficoll density gradient centrifugation in the laboratory following the manufacturer's instructions. The following cocktail of antibodies was used to stain PBMCs immediately after isolation to avoid the loss of immune epitopes caused by freeze–thaw: CD3 APC-H7, CD4 FITC, CD8 APC, CD45RA BV711, CCR7 BV650, PD-1 PE-Cy7, Tim-3 PE, and FVS510 AmCyan (all from BD Biosciences, USA). Within 24 h, cell-surface receptor expression was quantified by flow cytometry (FACS LSRFortess, BD, USA). At least 10^5^ cell populations were acquired as gated events and collated for each sample. Subsets analysis was conducted using Flowjo software (version 10.0, BD, USA). Appropriate isotype-matched controls or fluorescence minus ones were run in parallel for each sample.

### Quantitative polymerase chain reaction (qPCR)

Viral loads were observed using qPCR (Cobas Amplicor, Roche, Switzerland). TIANamp Genomic DNA kit (TIANGEN, China) was used to extract genomic DNA from PBMCs. Real-time qPCR amplification of sjTREC was performed following the method outlined by Douek et al. [13] Tsingke corporations, Shanghai, provided technical support for the plasmid of the standard sample and the validation of the qPCR products using Sanger sequencing. Quantification of sjTREC copies was evaluated using a standard curve, and highly conserved RAG2 gene expression was measured to calculate the intracellular copy numbers of sjTREC. Primers and TaqMan fluorescence probe were designed as follows: 1) sjTREC: forward primer 5’-ccatgctgacacctctggtt-3’; reverse primer 5’-tcgtgagaacggtgaatgaag-3’; fluorescence probe 5’-FAM-cacggtgatgcataggcacctgc-TAMRA-3’; 2) RAG2: forward primer 5’-tga agatgatactaatgaagagcagaca-3’; reverse primer 5’-cagagtcttcaaagggagtggaa-3’; fluorescence probe 5’-FAM-cccctggatcttctgttgatgtttgactgttttg-TAMRA-3’.

### Safety evaluation

The safety evaluation encompassed a review of clinical adverse events, electrocardiogram tests, and laboratory tests (blood tests, urine tests, renal and liver function, blood glucose, blood electrolyte, blood lipids, serum amylase, lipase, and cardiac muscle zymogram). Adverse events were evaluated according to the National Cancer Institute (NCI) Common Terminology Criteria for Adverse Events (CTCAE) version 5.0. This study is registered with ClinicalTrials.gov first time on 15/07/2021 as registration number NCT04963712.

### Statistical analysis

The data are represented as median and interquartile range (IQR). Multiple comparisons were performed by One-way Repeated Measures Anova (RANOVA) test or Friedman test based on its distribution tested by Shapiro–Wilk test. In cases where the *p*-value for multiple comparisons was less than 0.05, two-by-two comparisons between W4/W8/W12/W24 and W0 were conducted to obtain Bonferroni-adjusted *p*-values. For lost-to-follow-up data, the last assessable outcome was utilize as the follow-up endpoin. The statistical analysis was using SPSS software (version 13.0, IBM, USA). GraphPad Prism (version 7.0, GraphPad Software, USA) was used for visualizatio and plotting.

## Results

### Demographic and baseline characteristics

We evaluated 23 INRs for eligibility, of whom three were excluded due to an undetectable viral load for less than 2 years and withdrawal of informed consent prior to the initiation of the first dose. Consequently a total of 20 INRs were enrolled and received Tα1 treatment. The demographic and baseline characteristics of the participants are listed in Table 1. 95% of them were male, aged 48.1 (40.5, 57.0), and had received ART for 5 (3, 7.3) years. The nadir CD4 + T cell count was 22.0 (11.0, 109.0) cells/µL, with a corresponding CD4/CD8 T-cell ratio of 0.06 (0.04, 0.18) before ART. The CD4 + T cell count at baseline was 215.3 (190.1, 269.2) cells/µL, and CD4/CD8 T-cell ratio was 0.35 (0.24, 0.43). During the study, three participants withdrew their consents because of increased participation costs such as relocation or travel. Ultimately, 17 participants accomplished the protocol (Figure S1).

**Table 1:** Demographics and baseline characteristics of participants

Characteristic	*N* = 20 (%)
Sex	
Male	19 (95%)
Female	1 (5%)
Median age, years (IQR)	48.1 (40.5, 57.0)
Nadir CD4 + T cell count (IQR, cells/μL)	22.0 (11.0, 109.0)
Nadir CD4/CD8 T-cell ratio (IQR, cells/μL)	0.06 (0.04, 0.18)
Viral load, > 2 years (copies/mL)	< 50 (100%)
ART duration, years (IQR)	5.0 (3.0, 7.3)
Baseline CD4 + T cell count (IQR, cells/μL)	215.3 (190.1, 269.2)
Baseline CD4/CD8 T-cell ratio (IQR, cells/μL)	0.35 (0.24, 0.43)
ART regimens	
NRTIs + NNRTI	14 (70%)
NRTIs	3 (15%)
NRTIs + PI	2 (10%)
NRTI + INSTI	1 (5%)
Past history	
Hyperlipidemia	6 (30%)
Syphilis	3 (15%)
Hypercholesterolemia	2 (10%)
Hypertension	2 (10%)
Uarthritis	2 (10%)
Diabetes	1 (5%)
Renal insufficiency	1 (5%)
Hepatitis C infection	0 (0%)

*ART* antiretroviral therapy, *NRTI* nucleoside reverse transcriptase inhibitor, *NNRTI* non-nucleoside reverse transcriptase inhibitor, *PI* protease inhibitor, *INSTI* integrase strand transfer inhibitors, *IQR* interquartile range

### Safety evaluation

Twenty subjects received at least one dose of Tα1 injection. No severe drug-related adverse events were observed over the study period. The HIV viral load remained consistently stable and below the minimum detection level. Among the reported adverse events, five were deemed potentially drug-related, including hyperlipidemia, hypercholesterolemia, uarthritis, renal dysfunction, and sore throat (Table 2).

**Table 2:** Summary of adverse events

Preferred terms	All events	Drug-associated likely
**Abnormal indicators**	**62 (100%)**	**6 (9.6%)**
Hyperlipidemia	24 (38.7%)	1 (1.6%)
Syphilis	1 (1.6%)	0
Hypercholesterolemia	8 (12.9%)	1 (1.6%)
Hypertension	3 (4.8%)	0
Uarthritis	9 (14.5%)	1 (1.6%)
Diabetes	9 (14.5%)	0
Renal insufficiency	6 (9.8%)	1 (1.6%)
Upper respiratory tract infection	1 (1.6%)	1 (1.6%)
Sore throat	1 (1.6%)	1 (1.6%)

### T cell counts

**Fig. 1:**
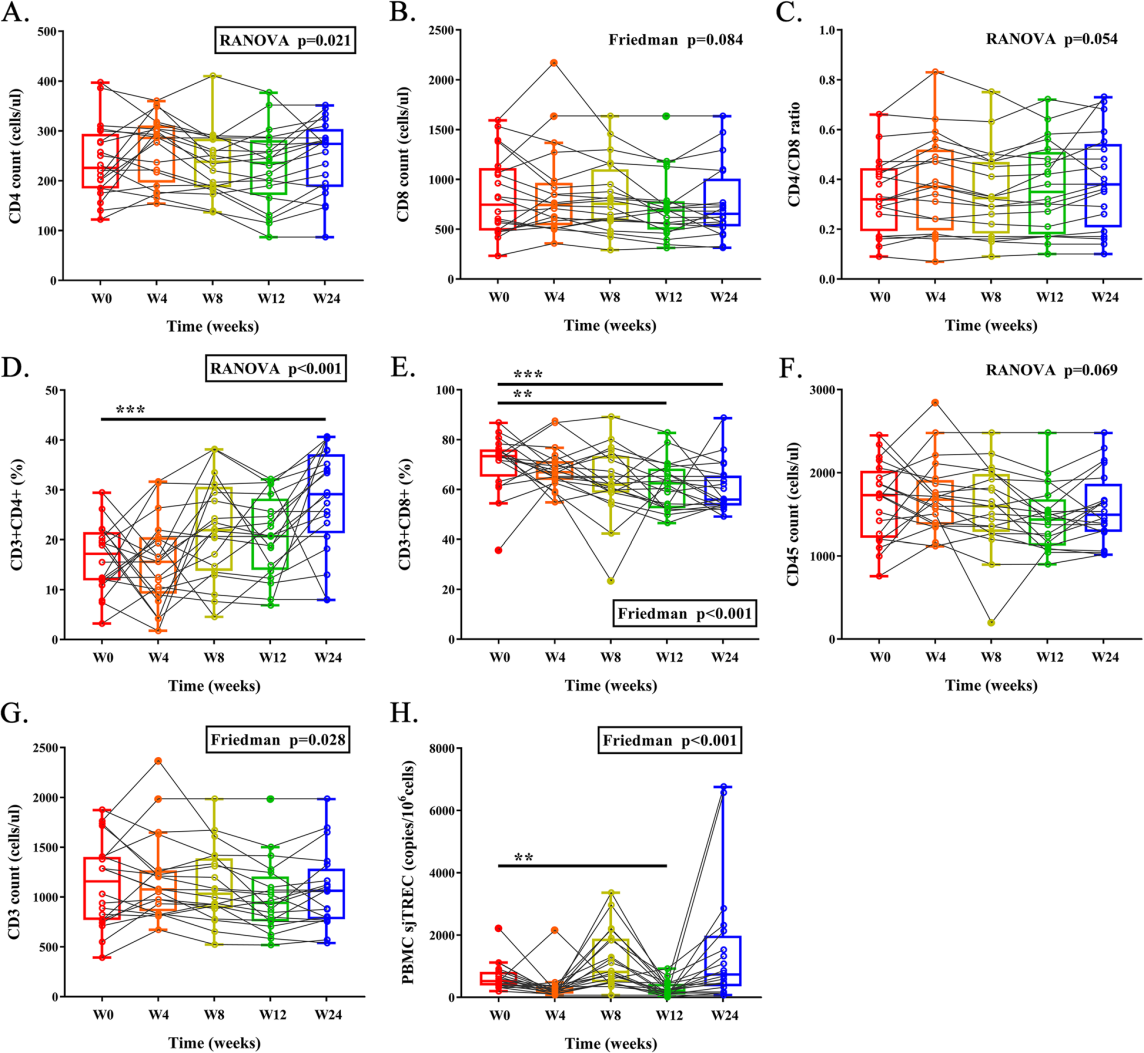
Lognitudinal changes in CD4 + T cell count (**A**), CD8 + T cell count (**B**), CD4/CD8 T-cell ratio (**C**), proportion of CD3 + CD4 + T cell (**D**), proportion of CD3 + CD8 + T cell (**E**), CD45 + lymphocyte count (**F**), CD3 + cell count (**G**), and PBMC sjTREC (**H**). PBMC: peripheral blood mononuclear cell; sjTREC: signal joint T cell receptor excision circles

Lognitudinal changes in CD4 + T cell count, CD8 + T cell count, CD4/CD8 T-cell ratio, CD45 + lymphocyte count, CD3 + T cell count, and proportions of CD3 + CD4 + or CD3 + CD8 + T cell subsets within 24 weeks were depicted in Fig. 1. Multiple comparisons revealed significant overall changes in CD4 + T cell count, but no difference was observed between baseline and week 24 (Fig. 1A). The CD4/CD8 T-cell ratio and CD8 + T cell count had not change at week 24 and overall (Fig. 1B, C). The proportion of CD3 + CD4 + T cells increased (17.2% vs. 29.1%, *P* < 0.001; Fig. 1D), while CD3 + CD8 + T cells decreased (73.3% vs. 56.0%, *P* < 0.001; Fig. 1E). No significant changes were observed in total CD45 + lymphocyte count and CD3 + T cell count between baseline and week 24. (Fig. 1F, G).

### Thymic output measurement

Tα1 promotes the proliferation, differentiation and maturation of T cells in the thymus. Based on the level of PBMC sjTREC, we calculated the degree of thymic output. The level of PBMC sjTREC had trend of increase but no significance at week 24 in comparison to that at baseline (521.2 copies/10^6^ cells vs. 753.3 copies/10^6^ cells) (Fig. 1H). Thymic output was altered overall (*p* < 0.001) while a significant decrease was observed at week 12 (521.2 copies/10^6^ cells vs. 216.4 copies/10^6^ cells, *P* = 0.008). One possible hypothesis is that the participants with impaired thymic function were astatically hyposensitive to Tα1 treatment at that time.

### T cell subsets and immune checkpoints

**Fig. 2:**
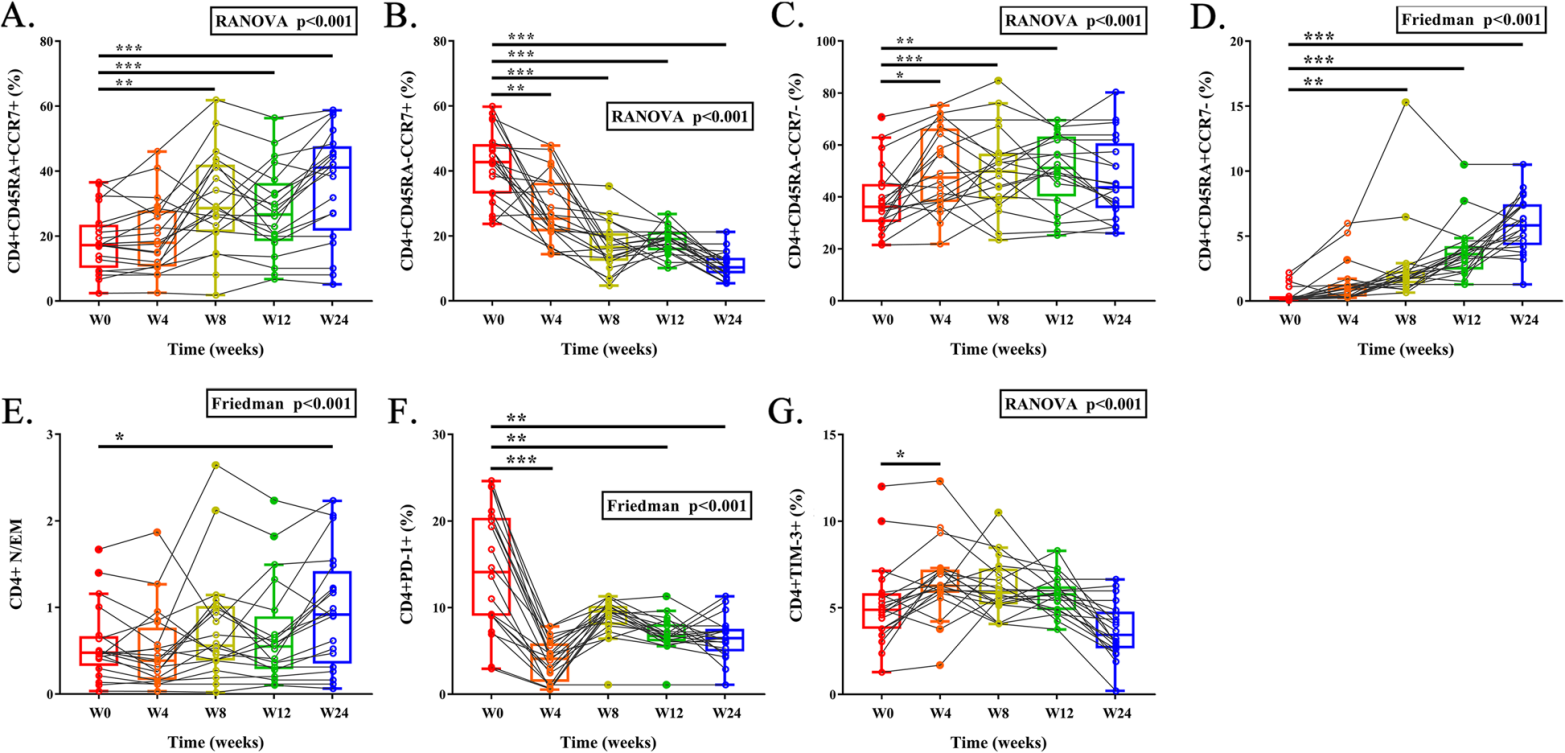
Longitudinal changes of proportions of CD4 + naive T cell (**A**), central memory T cell (**B**), effector memory T cell (**C**), TEMRA T cell (**D**), N/EM T cell ratio (**E**), and corresponding expression of immune checkpoints (**F**, **G**). N/EM: central memory/effector memory. TEMRA: terminal effector memory CD45RA re-expressing

**Fig. 3:**
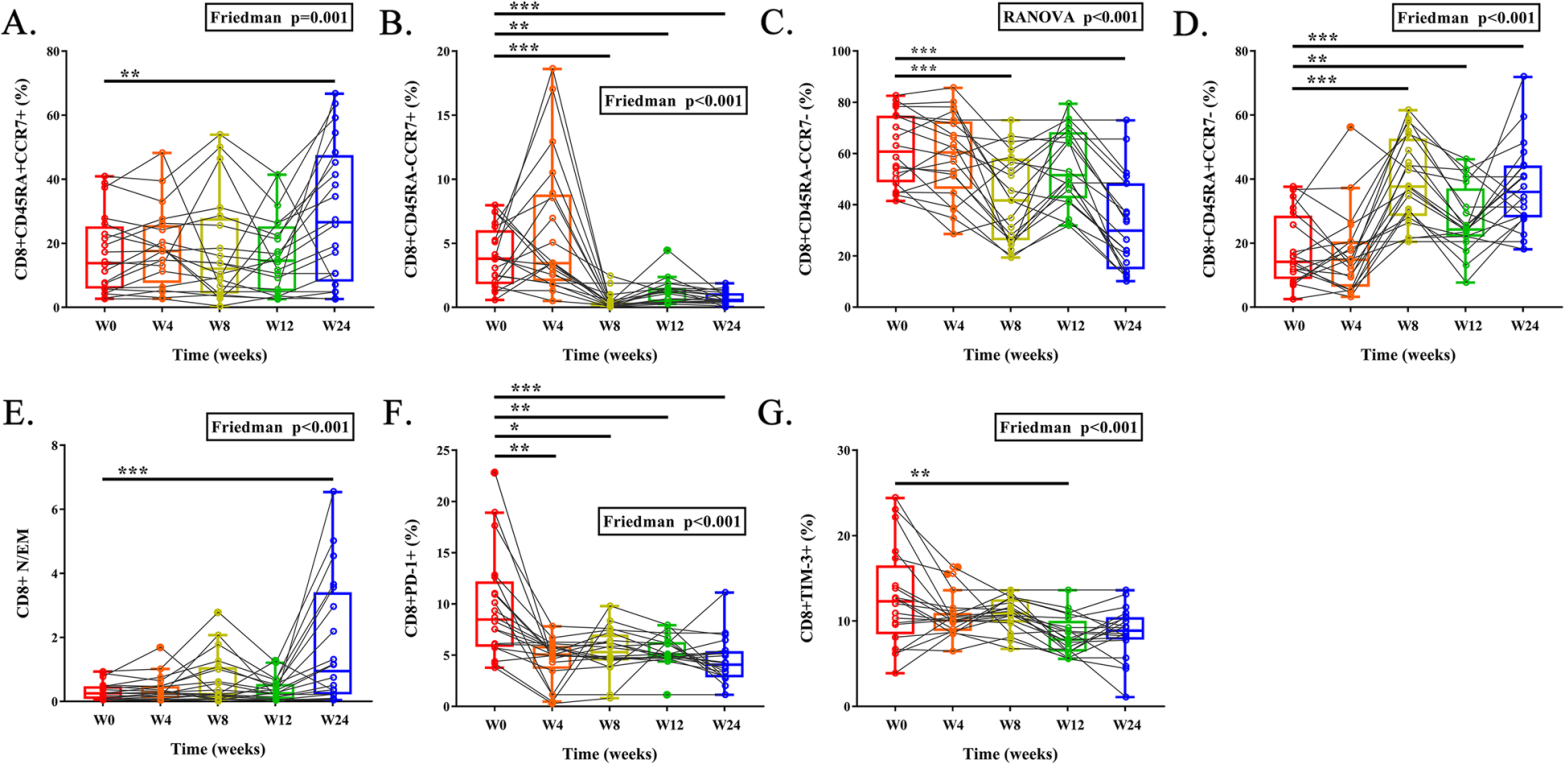
Longitudinal changes of proportions of CD8 + naive T cell (**A**), central memory T cell (**B**), effector memory T cell (**C**), TEMRA T cell (**D**), N/EM T cell ratio (**E**), and corresponding expression of immune checkpoints (**F**, **G**)

In Figs. 2A and 3A, both proportions of naïve (CD45RA + , CCR7 +) CD4 + T cell (17.2% vs. 41.1%, *P* < 0.001) and naïve CD8 + T cell (13.8% vs. 26.6%, *P* = 0.008) increased at week 24 compared to that at baseline. In contrast,, the proportions of central memory (CM, CD45RA-, CCR7 +) CD4 + T cell (42.7% vs. 10.3%, *P* < 0.001) and CM CD8 + T cell (3.8% vs. 0.6%, *P* < 0.001) both decreased (Figs. 2B and 3B). The proportion of effector memory (EM, CD45RA-, CCR7-) CD4 + T cell had no change, but the proportion of EM CD8 + T cell (60.8% vs. 29.9%, *P* < 0.001) sharply decreased (Figs. 2C and 3C). Additionally, proportions of terminal effector memory (TEMRA, CD45RA + , CCR7-) CD4 + T cell (0.2% vs. 5.8%, *P* < 0.001) and TEMRA CD8 + T cell (14.2% vs. 36.0%, *P* < 0.001) increased (Figs. 2D and 3D). In light of the notable changes in T cell subsets, the naïve/effector memory CD4 + T cell ratio (N/EM ratio) and the CD8 + N/EM ratio gained great increases at week 24 (0.48 vs. 0.92, *P* = 0.014, Fig. 2E and 0.25 vs. 0.95, *P* < 0.001, Fig. 3E).

The expression of immune checkpoints such as programmed cell death 1 (PD-1) and T cell immunoglobulin and mucin domain-containing molecule-3 (Tim-3) delineates the degree of immune exhaustion. In comparison to baseline, the proportions of CD4 + T cells and CD8 + T cells that expressed PD-1 significantly decreased after Tα1 treatment (CD4 + PD-1 + , 14.1% vs. 6.5%, *P* = 0.002; CD8 + PD-1 + , 8.5% vs. 4.1%, *P* < 0.001) at multiple time-points (Figs. 2F and 3F). Furthermore, the proportions of CD4 + T cells and CD8 + T cells expressed TIM-3 exhibited a decreasing trend after Tα1 treatment, however, did not reach statistical significance (CD4 + TIM-3 + , 4.9% vs. 3.4%; CD8 + TIM-3 + , 12.3% vs. 8.9%, Figs. 2G and 3G). The gate strategy and representative diagram of flow cytometry sorting were shown in Figure S2.

## Discussion

In spite of the mounting life expectancy of PLWH after ART treatment worldwide, INRs living with HIV were still suffering from severe conditions due to diminished thymic output, bone marrow hematopoiesis dysfunction, residual virus replication, immune exhaustion, and aberrant immune activation [6, 7]. Impaired thymic function in patients with low CD4 + T cell counts may account for inadequate CD4 + T cell restoration [14]. In this study, Tα1 treatment altered CD4 + T-cell counts and thymic output characterized by PBMC sjTREC at an overall level after initiation. Previous studies have demonstrated a correlation between levels of PBMC sjTREC, recent thymic emigration, long-time CD4 + T count response, and survival in PLWH [13, 15, 16]. However, more in-depth investigations have revealed that the elevation of sjTREC levels could be the consequence of homeostatic proliferation of CD45RA + naïve T cells in the periphery [17, 18]. Combined with the great increase of naïve T cells in this study, the use of sjTREC as a measure of thymic output might carry the risk of producing false-positive results. In contrast, the sj/βTREC ratio, serving as a surrogate for thymic output, remained unaffected by peripheral proliferation and predicted the therapy-mediated recovery of naive and total CD4 + T cells, as well as HIV disease progression [19, 20]. Given the positive correlation between the sj/βTREC ratio and CD4 + naïve T cells as demonstrated (*R* = 0.504, *P* = 0.009 in Dion ML, Blood 2007), the findings of increased thymic output in this study still hold merit.

Traditionally, the CD4 + T cell count and the CD4/CD8 T-cell ratio serve as primary indicators for evaluating immune reconstitution in PLWH [21]. We observed a difference in multiple comparisons of CD4 + T cell counts and a gradual, albeit insignificant, increase in the median CD4 + T cell count throughout the treatment period (225.8 vs. 286.0 vs. 238.1 vs. 236.0 vs. 274.0). Although a large gap still existed from satisfactory level (> 350 cells/μL), we had reason to believe that participants achieve an improved immune profile and prognosis. The feasibility of the N/EM T cell ratio as a useful predictive marker of immune reconstitution in chronic HIV patients suggested the role of Tα1 in immune reconstitution [22]. In brief, our results suggest that INRs achieve partial immune reconstitution and probably a better prognosis after treatment.

We confirmed that Tα1 was safe and well tolerated in this group, consistent with previous clinical trials of Tα1 in PLWH [10–12]. While different immunomodulating compounds like IL-2 were used to restore CD4 + T cell count in HIV infection, they failed to reduce the incidence of opportunistic disease or death because the expanded CD4 + T cells lacked host defense capacity. [23] Encouragingly, recombinant human IL-7 was proven well tolerated and result in a dose-dependent increase of CD4 + T cell and thymic output in patients with poor immune reconstitution [24]. Considering that Tα1 has demonstrated its effectiveness in elevating CD4 + T cells after 12 months [10], a larger and longer cohort study is necessary to further investigate the therapeutic efficacy of Tα1 for INRs living with HIV in the future.

Immunophenotyping analysis revealed the distinct roles played by T cell subsets at various stages of development. The production of de novo naïve T cells in the thymus has been demonstrated to push reconstitution of all T cell subsets [14]. Based on our findings, Tα1 reversed the decrease of proportions of naïve T cells in chronic HIV infection, and reduced the proportions of exhausted PD-1 + T cells caused by sustained abnormal immune activation. Besides, it significantly reduced the proportion of CM CD4 + T cells, which have been identified as primary host cells for HIV reservoir [25]. Fromentin et al. found that CD4 + CM T cells co-expressing PD-1 and CTLA-4 exhibit diminished responsiveness to activating stimuli facilitating HIV latency, and the PD-1/PD-L1 inhibitor Pembrolizumab induced HIV latency reversal in PLWH [26, 27]. HIV persists preferentially in CD4 + T cells expressing multiple immune checkpoint molecules, including PD-1, TIM-3, CTLA-4 and LAG-3. Therefore, combining immune checkpoints to reverse latency is a more effective strategy than using a single latency reversal agent, like vorinostat and bryostatin [28]. In fact, some studies found INRs maintain a lower proportion of thymic migrated cells and naïve T cells, a higher proportion of CD4 + PD-1 + T cells, and a higher level of HIV DNA and CA-RNA compared to IRs [7]. Mechanistically, HIV could evade host immune responses by upregulating the expression of immune checkpoint receptors, like PD-1, CTLA-4, and Tim-3 [29]. Our findings extend one underlying scientific hypothesis that Tα1 treatment exerts a positive effect on the regeneration of T-cell storage, and participated in diminishing the HIV reservoir, which represents the focal point of our forthcoming investigations.

The present study has some limitations. First, the sample size is relatively small. Thus, a larger cohort is required to confirm the safety and efficacy of Tα1 on INRs living with HIV. Second, the absence of a control group, such as a placebo arm. Third, the utilization of sjTREC rather than sj/βTREC ratio as a surrogate for thymic output might introduce some degree of false-positive results. Lastly, longer duration of treatment might be necessary to achieve statistically significant increases in CD4 + T cell counts.

## Conclusions

In summary, we first report that Tα1 treatment for INRs living with HIV was well-tolerated. Tα1 treatment improves the CD4 + T cell count at overall level but not at endpoint. Notably, Tα1 increases thymic output, rebuilds the composition of T cell subsets, and partially reverses immune exhaustion. The efficacy of Tα1 treatment in INRs necessitates further evaluation through extended and expanded clinical trials, as well as comprehensive mechanistic investigations.

### Supplementary Information

**Additional file 1: Figure S1.** Trial flow. Blue curved arrows represent loss to visit. Image drawn by figdraw online. INR: immunological nonresponder; ART: antiretroviral therapy; qd: quaque die; biw: biweekly.

**Additional file 2: Figure S2.** Gate strategy and representative diagram of flow cytometry sorting. CM: central memory; EM: effector memory; TEMRA: terminal effector memory CD45RA re-expressing.

## Data Availability

Shanghai Public Health Clinical Center, China is committed to data integrity and accuracy. Summary trial information for study NCT04963712 is available on ClinicalTrials.gov. The datasets generated during and/or analyzed during the current study are available from the corresponding author on reasonable request.

## Abbreviations

INRs: Immunological nonresponders
sjTREC: Signal joint T cell receptor excision circles
HIV: Human immunodeficiency virus
PBMCs: Peripheral blood mononuclear cells
PLWH: People living with HIV
ART: Antiretroviral therapy
IRs: Immunological responders
Tα1: Thymosin α1
PVB: Peripheral venous blood
qPCR: Quantitative polymerase chain reaction
NCI: National Cancer Institute
CTCAE: Common Terminology Criteria for Adverse Events
IQR: Interquartile range
TEMRA: Terminal effector memory
N/EM ratio: Naïve/effector memory ratio
PD-1: Programmed cell death 1
Tim-3: T cell immunoglobulin and mucin domain-containing molecule-3
