# Supplementary material for: Role of thymosin α1 in restoring immune response in immunological nonresponders living with HIV
Source: BMC Infect Dis. 2024 Jan 17;24:97. doi: 10.1186/s12879-024-08985-y (PMC10792804; doi:10.1186/s12879-024-08985-y)
Supplement: Supplementary file 1 — Additional file 1: Figure S1. Trial flow. Blue curved arrows represent loss to visit. Image drawn by figdraw online. INR: immunological nonresponder; ART: antiretroviral therapy; qd: quaque die; biw: biweekly. [file 12879_2024_8985_MOESM1_ESM.docx]

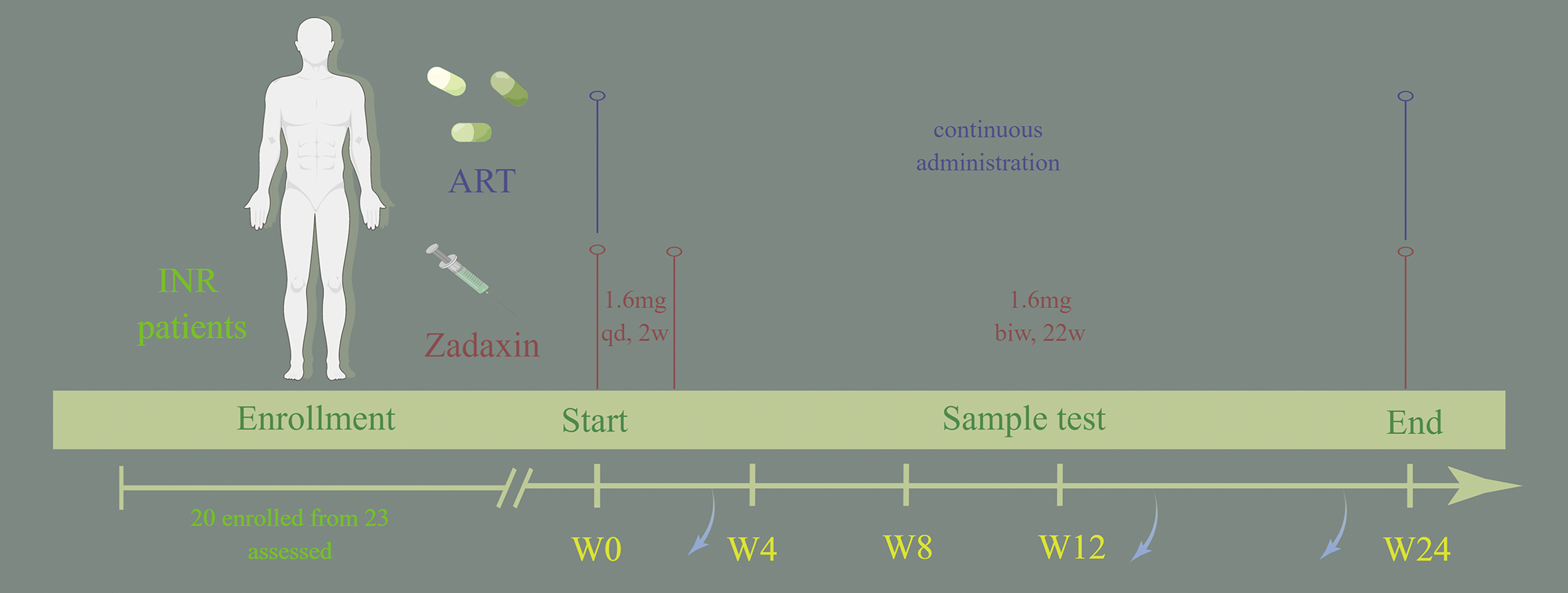


**Figure S1. Trial flow.** Blue curved arrows represent loss to visit. Image drawn by figdraw online. INR: immunological nonresponder; ART: antiretroviral therapy.
