# Supplementary figures and images for: Role of thymosin α1 in restoring immune response in immunological nonresponders living with HIV
Source: BMC Infect Dis. 2024 Jan 17;24:97. doi: 10.1186/s12879-024-08985-y (PMC10792804; doi:10.1186/s12879-024-08985-y)

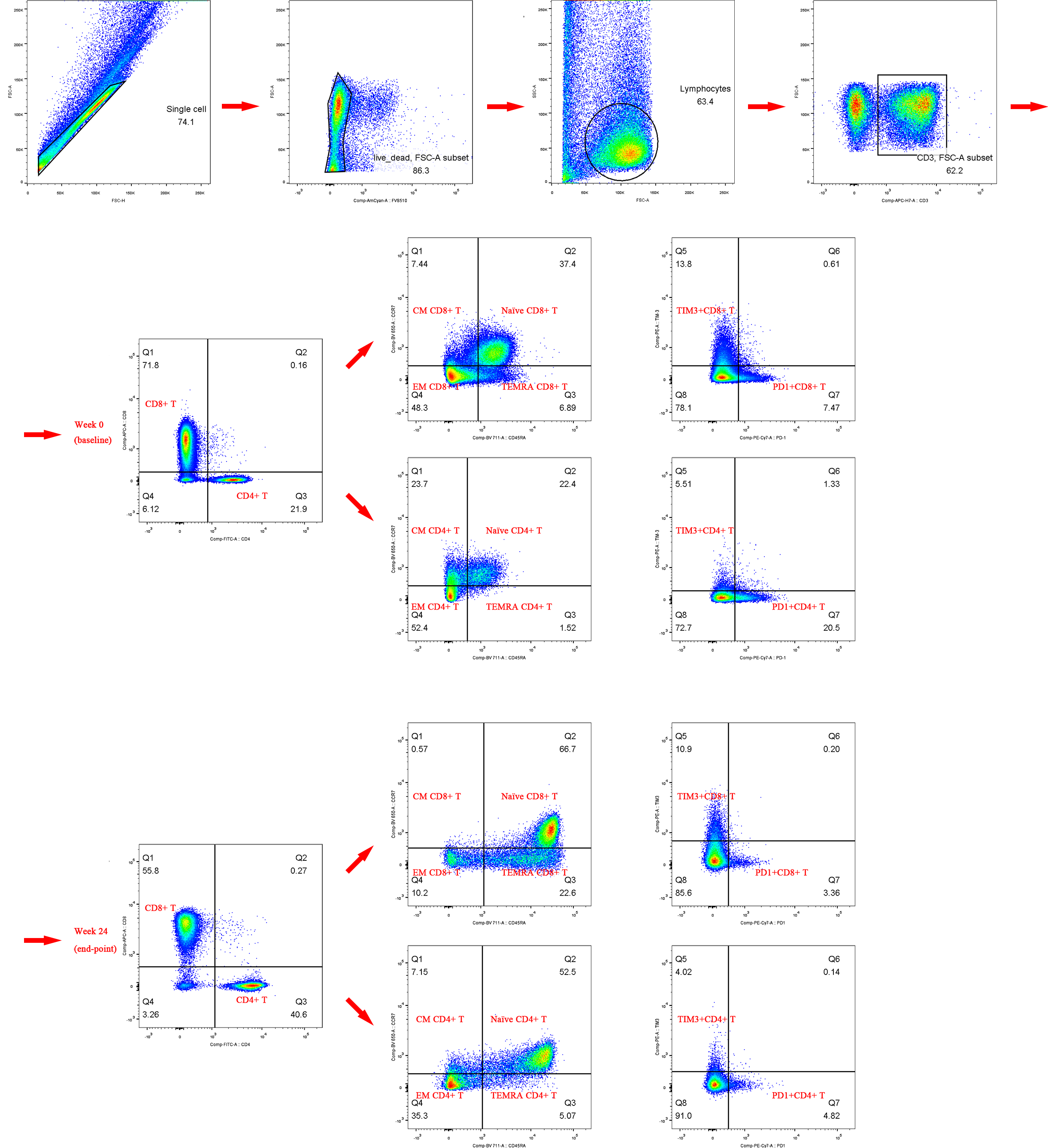

Supplement: Supplementary file 2 — Additional file 2: Figure S2. Gate strategy and representative diagram of flow cytometry sorting. CM: central memory; EM: effector memory; TEMRA: terminal effector memory CD45RA re-expressing. [file 12879_2024_8985_MOESM2_ESM.tif]
